# Mechanical properties and microstructure of pre-treated luffa fiber reinforced cement mortar

**DOI:** 10.1371/journal.pone.0314213

**Published:** 2025-02-07

**Authors:** Renqiang Yang, Zhengjun Guan, Lihua Zhang, Yong Shu

**Affiliations:** 1 College of Engineering and Technology, Southwest University, Chongqing, China; 2 College of Smart Health, Chongqing Polytechnic University of Electronic Technology, Chongqing, China; 3 School of Mechanical and Electrical Engineering, Sichuan Agricultural University, Ya’an, China; Covenant University, NIGERIA

## Abstract

In this work, leveraging the enhanced wear resistance, toughness, and renewability of luffa fiber, pretreated luffa fiber was applied into cement mortar to investigate the impact of different fiber contents and sizes on mortar performance. Meanwhile, the fiber-mortar interface fusion and hydration products were systemically analysed by performing SEM-EDS (scanning electron microscopy-energy spectrum analysis) and CT (Computed Tomography) tomography measurements. From our analysis, it was demonstrated that pretreated luffa fibers could significantly enhance the strength, shrinkage resistance, and toughness of cement mortar. When the fiber content was 1% and the length was 1 cm, the 28-day compressive and flexural strengths of the cement mortar reached 57.63 MPa and 9.68 MPa, respectively, representing an increase of 10.81% and 9.47% compared to ordinary cement mortar. When the fiber content was 1%, with fiber lengths of 1 cm and 2 cm, the 56-day drying shrinkage rates of the cement mortar were 2.78% and 6.09%, respectively. This result corresponds to a reduction in shrinkage by factors of 7.17 and 3.27, respectively, compared to standard cement mortar. Additionally, with a fiber content of 1% and lengths ranging from 1 to 3 cm, the load-deflection behaviour of luffa fiber cement mortar was noticeably superior to that of conventional mortar. The SEM-EDS images revealed that cement mortar containing 1 cm fibers had a substantial presence of tinfoil-like C-S-H (hydrated calcium silicate) and needle-like AFt (ettringite) structures. In addition, better integration with the cement mortar compared to other fiber lengths was demonstrated. CT tomography showed that luffa fibers were concentrated in large amounts at the top and bottom of the test samples, with an increase in voids and fiber agglomeration as the fiber content increased. In summary, when the luffa fiber content was 1% and the fiber length was 1 cm, the mechanical performance of cement mortar could be effectively enhanced and fiber agglomeration can be mitigated, suggesting potential applications in building materials.

## 1. Introduction

### 1.1 Status and content of current research

In recent years, driven by urbanization, China’s construction area has maintained an average annual growth rate ranging from 3% to 5%. At present, CO_2_ emissions from China’s construction industry reach about 2.1 billion tons, constituting 20% of the national CO_2_ emissions [1, 2]. To promote sustainable development, "carbon peaking and carbon neutrality" must be integrated into the overall framework of ecological civilization. The use of renewable resources in the construction industry will help achieve sustainability [3, 4]. However, ordinary concrete and cement mortar exhibit low tensile strength, poor toughness, easy cracking, and low impermeability. Particularly, in reinforced concrete, chloride ions can easily react with the steel reinforcement, causing corrosion, which compromises the durability of the concrete structure [5–8]. As a low-cost and renewable polymer material, plant fibers, when applied in cement mortar or concrete, can significantly improve their crack resistance and durability, while also aiding in the recycling of agricultural waste resources [9–11]. The use of plant fiber-reinforced mortar or concrete has emerged as a key trend for future development [12–14].

Luffa, derived from the mature fruit of luffa plants, constitutes a three-dimensional porous network of interwoven filamentous fibers, known for its natural attributes including high mechanical strength, lightweight, wear resistance, toughness, and elasticity [15, 16]. It is widely applied in various fields, such as biology, environment, and materials science [17–20]. For instance, Nie et al. developed the (LF-A2-M1/P) adsorbent by grafting amide and phosphate groups onto the surface of the luffa fiber via chemical methods. Thermodynamics indicated that the adsorption of U(VI) by LF-A2-M1/P is a spontaneous process, with a maximum adsorption capacity of 353.85 mg/g [21]. Feng et al. synthesized MnOx/luffa fiber composites by catalytically oxidizing the luffa fiber with transition metal oxides, demonstrating excellent formaldehyde removal performance at room temperature [22]. Lin et al. attached fluorinated silica nanoparticles (F-SiO_2_NPs), prepared by the sol-gel method, on the surface of the luffa fiber to create a novel environmentally friendly three-dimensional superhydrophobic material [23]. In another interesting work, Salih et al. utilized luffa fiber and urea formaldehyde resin to manufacture insulation layer panels, which maintained their performance even in hot and dry conditions. When exposed to high temperatures, the panels exhibited a K value increase to 0.26W/mK, indicating effective insulation properties [24]. Although luffa fiber has been applied in fields such as biology, environment, and materials, its implementation in cement mortar and concrete is relatively scarce. Common plant fibers, such as rice husk, coconut shell, sisal, pine needles, and jute, have already been successfully used in cement mortar and concrete as building materials [25–29]. For example, Zhang et al. incorporated jute fiber into concrete and tested the compressive and tensile strengths of jute fiber concrete specimens at 28 days of curing, discovering that jute fiber can significantly enhance concrete toughness, crack resistance, and the tensile-to-compressive strength ratio of concrete, while also determining the optimal fiber length and mixing ratio [30]. Long et al. found that by adding 0.5%, 1%, 1.5%, and 2% of pretreated pine needle fibers, the compressive strength, splitting tensile strength, and fracture modulus of the concrete could be improved, as well as the ductility and toughness of the specimens [31]. In addition, Bheel et al. investigated the mechanical properties of concrete by adjusting the content of jute fiber and straw ash. The authors reported that the appropriate combination of jute fiber and straw ash could significantly enhance the compressive and splitting tensile strengths of concrete [32]. However, these works also revealed certain limitations when using rice husk, coconut shell, and sisal in building materials. For instance, the majority of the reported works in the literature on plant fiber-reinforced cement-based composites focus on their physical and mechanical properties, while the targeted and practical modification methods for plant fibers still need improvement. Particularly, the flowability, shrinkage performance, and interface fusion issues between the plant fibers and cementitious materials have been studied to a limited extent.

Simultaneously, limited research has been also conducted on the application of luffa fibers in construction materials and certain constraints remain. For instance, Abd-Al Ftah et al. incorporated only 0.75% luffa fibers into concrete to examine the mechanical ultimate load, load-deflection relationship, and stiffness, deriving some conclusions [18]. However, the authors did not pre-treat the luffa fibers, as the presence of numerous impurities in the fibers impeded the setting and hardening process of concrete. Additionally, the impact of different fiber sizes and dosages of luffa fibers on the mechanical properties of concrete was not taken into account, and the interaction between the luffa fibers and the concrete matrix was not in detail analysed. Likewise, Anandaraj et al. explored the mechanical performance of luffa fiber-reinforced concrete containing rice husk ash, noting that 3% and 4% luffa fibers had adverse effects on the concrete properties [19]. The authors further asserted that a combination of 1% luffa fibers with 25% rice husk ash yielded the best compressive, splitting tensile, and flexural strengths. However, Anandaraj’s study also presented limitations, as it did not address the influence of luffa fibers on the flowability, shrinkage, and microstructure of cementitious materials. On top of that, the provided interpretations regarding the mechanical properties of luffa fibers in concrete lacked depth and theoretical rigor.

To effectively deal with these issues and consider the enhanced mechanical properties of luffa fibers, such as high toughness, elasticity, and lightweight characteristics [33, 34], the limitations of the previously reported works in the literature on plant fiber-reinforced concrete were addressed here. More specifically, a 2% NaOH pretreatment was applied to luffa fibers to disrupt the hydrogen bonds within the fiber structure and depolymerize polysaccharides. Thereby, the surface roughness of the fibers was increased and the interfacial bonding between the fibers and the cement mortar was improved. Additionally, the impact of 1%-3% fiber content and 1 cm-3 cm fiber length on the fluidity, flexural strength, compressive strength, drying shrinkage, and deflection of the cement mortar were thoroughly investigated. Furthermore, SEM-EDS and CT tomography measurements were used to analyse the interface fusion and hydration products between the luffa fibers and the cement mortar, verifying the feasibility of the luffa fiber-reinforced cement mortar.

### 1.2 Research significance and objectives

Luffa plant fibers are widely distributed in China, with an annual production of over 100 million tons. These fibers possess comparative advantages, such as light weight, wear resistance, toughness, and renewability. However, the application of luffa fibers in cement mortar has rarely been reported. Along these lines, in this work, leveraging the advantages of luffa fibers and the gaps in the existing works in the literature, they were applied in cement mortar to enhance various mechanical properties like compressive strength, flexural strength, drying shrinkage, and deflection. Additionally, our approach contributes to turning waste into treasure, achieving the goals of a circular economy and the "dual carbon" strategy. Ultimately, the luffa fiber-cement mortar can be promoted for use in building construction and road projects.

### 1.3 Advantages and limitations of our work

This work creatively applied luffa fibers to cement mortar, whereas the majority of the previously reported works in the literature mainly focused on applying luffa fibers to cement concrete and analysing its properties. Additionally, pre-treated luffa fibers in cement mortar were used, and the impact of different fiber lengths and dosages on the mechanical properties, shrinkage, deflection, and interface fusion of the mortar was systematically investigated. A more detailed analysis is also provided compared to earlier studies, highlighting the novelty of our work. However, several limitations will be the focus of future research, including the impact freeze-thaw resistance, alkali corrosion, and sulfate attack resistance of luffa fiber-cement mortar on the mechanical properties.

## 2. Materials and methods

### 2.1 Raw materials

**Fig 1:**
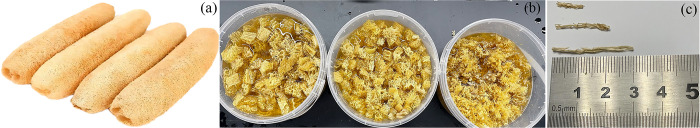
The original sample of luffa, 2%NaOH pre-treatment sample, and different sizes of luffa fibers.

The cement is 42.5 ordinary Portland cement purchased from Chongqing Hongshi Cement Factory, with a specific surface area of 3500 cm^2^/g and a density of 3.2 g/cm^3^. IOS standard sand is purchased from Xiamen Aisiou Standard Sand Co., Ltd. NaOH is an analytical reagent (AR) acquired from Tianjin Zhiyuan Chemical Reagent Co., Ltd. luffa comes from Chongqing, China, with a golden yellow appearance, a long cylindrical shape, a length of 30–70 cm, and a diameter of 7–10 cm. The density of luffa is 1.06 g/cm^3^, and the tensile strength is 200–700 MPa. Due to the high impurities and abundant organic matter on the surface of luffa fibers, NaOH treatment is required to disrupt the hydrogen bonds in the structure and depolymerize the hemicellulose to increase the surface roughness of luffa fibers. The latter is beneficial for enhancing the interfacial bonding between fibers and cement mortar [31, 35]. The pretreatment process involves immersing the luffa fibers in a 0.5 mol/L NaOH solution for 12 hours at a room temperature of 20°C, followed by rinsing and air-drying the soaked luffa fibers to remove excess surface moisture. Fig 1(A)–1(C) show the original sample of luffa material, the pre-treatment sample soaked in 2% NaOH for 12 hours, and the different sizes of luffa after pre-treatment.

### 2.2 Mix design

The dosage values of luffa fiber were 0.5%, 1%, 2%, and 3% (Based on cement admixture), and the lengths of luffa fiber were 1 cm, 2 cm, and 3 cm, respectively. The dosage of cement, water, and sand shall be 450 g, 1350 g, and 225 g according to GB/T 17671–2021 "Testing Method for Strength of Cement Mortar" [36]. The specific cooperation is listed in Table 1 below.

**Table 1:** Mix proportions of luffa fiber reinforced cement mortar.

Number	Cement content/g	luffa size/cm	luffa content/%	Standard sand content/g	water/g
A0	450	/	/	1350	225
A1	450	1	0.5%	1350	225
A2	450	1	1%	1350	225
A3	450	1	2%	1350	225
A4	450	1	3%	1350	225
B1	450	2	0.5%	1350	225
B2	450	2	1%	1350	225
B3	450	2	2%	1350	225
B4	450	2	3%	1350	225
C1	450	3	0.5%	1350	225
C2	450	3	1%	1350	225
C3	450	3	2%	1350	225
C4	450	3	3%	1350	225

### 2.3 Preparation process of luffa fiber reinforced cement mortar specimens

**Fig 2:**
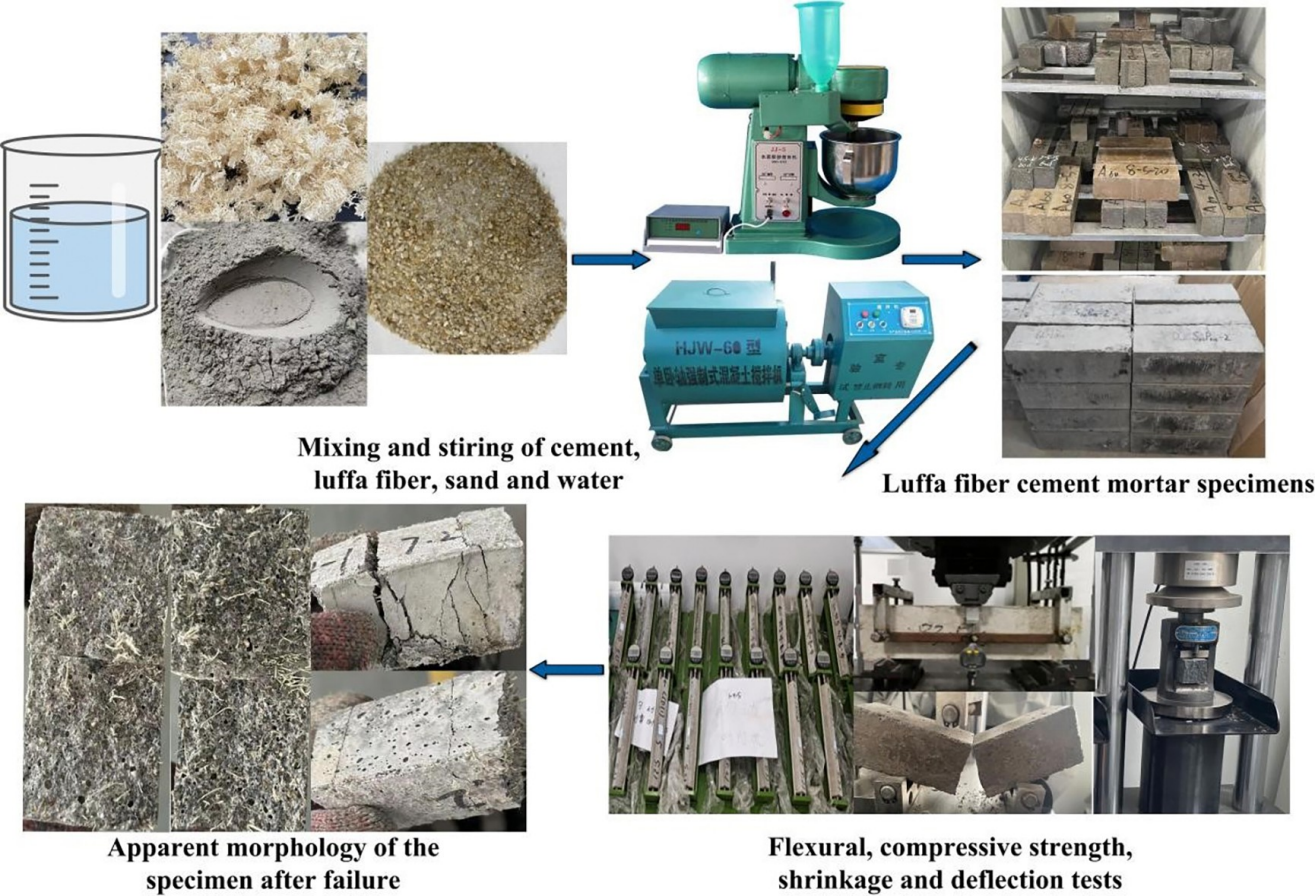
Preparation process of luffa fiber reinforced cement mortar.


Mortar specimens with dimensions of 40 mm×40 mm×160 mm were prepared according to GB/T 17671–2021 "Methods for Testing the Strength of Cement Mortar" [36]. The prepared luffa fiber was pre-mixed with cement for 2 minutes, and then, standard sand and water were added and thoroughly mixed in the mixer. The mixture was next injected into the mold and vibrated. The formed mortar specimens were demolded after being cured in the curing box at 20±2°C and 95% humidity for 24 hours, followed by additional curing until 3 days, 7d days, and 28 days for subsequent mechanical testing and characterization. According to JC/T 603–2004 "Standard Test Method for Drying Shinkage of Mortar", the dry shrinkage of luffa fiber reinforced cement mortar was tested. The cement to standard sand ratio in the mortar was 1:2 (mass ratio), the water cement ratio was 0.5, and the specimen size was 25 mm×25 mm×280 mm. The length of luffa fiber reinforced cement mortar was measured at 3, 7, 14, 21, 28, and 56 days, and the formula for calculating the drying shrinkage rate ɛ is shown in Eq (1) [37]. According to the American ASTM C1018 specification, the load-deflection curve was obtained [38]. According to the specification, the size of the luffa fiber reinforced cement mortar was 100 mm×100 mm×400 mm and the span was 300 mm. For the CT scan measurements, a cylindrical specimen with a diameter of 100 mm and a height of 120 mm was used. The preparation process of luffa fiber reinforced cement mortar is shown in Fig 2.

$$\varepsilon =(L_{0}‐L_{t})/L_{0}$$ (1)

In the formula ɛ denotes the drying shrinkage rate of mortar at t day, L_0_ is the measured length of the mortar specimen on the first day, mm, and L_t_ refers to the measured length of the mortar specimen at t days, mm.

### 2.4 Analysis and characterization methods

Using a cement concrete compression testing machine (YAW-300D), compressive and flexural strength tests were conducted on luffa fiber reinforced cement mortar at a displacement rate of 2.4 KN/s. The load-deflection test was carried out using a microcomputer controlled electronic universal testing machine (MTS-E64.206, 2000kN) with a speed of 0.1 mm/min. During the test, the computer automatically collects data to obtain the load-deflection curve of the specimen. A thin sample from the crushed hardened body was taken and immersed in anhydrous ethanol for 48 hours to stop hydration. Next, gold was sprayed on the thin sample and its microstructure was observed using a scanning electron microscope (SEM) and energy dispersive spectroscopy (EDS) (Zeiss Sigma-300). The CT scanning experiment used Multiscale Voxel Industrial CT (MS Voxel450) produced by Tianjin Sanying Precision Instrument Co., Ltd. to scan cylindrical luffa fiber reinforced cement mortar.

## 3. Analysis and discussion

### 3.1 Luffa fiber mortar flowability

**Fig 3:**
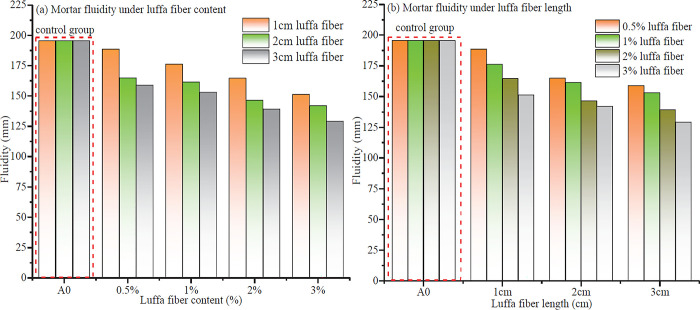
Luffa fiber mortar flowability.

Fig 3 shows the flowability of mortar with different luffa fiber content and lengths. As can be observed from Fig 3(A), when the length of luffa fibers was the same, the flowability of the mortar gradually decreased with an increasing amount of added luffa fibers. Taking a length of 1 cm as an example, the flowability of cement mortar with a luffa fiber content of 0%, 0.5%, 1%, 2%, and 3% corresponds to 195.5, 188.6, 176.2, 164.6, and 151.2 mm, respectively. In general, under the same length conditions, the flowability of cement mortar without luffa fibers was greater than that of mortar with added fibers. Besides, as the fiber content increased, the flowability of the mortar decreased. This outcome indicates that luffa fibers have a relatively high water absorption capacity, and the incorporation of luffa fibers reduces the flowability of mortar. Fig 3(B) indicates that with the increase in the length at the same amount of luffa fiber content, the flowability of luffa fiber cement mortar decreased. Taking the luffa fiber content of 0.5% as an example, when the lengths of luffa fibers were 0, 1, 2, and 3, and the corresponding flowability of luffa fiber cement mortar was 195.5, 188.6, 164.9, 158.8 mm, respectively. Under the same luffa fiber content, shorter lengths result in greater flowability of mortar. This effect stems from the formation of networks curls, and entanglements inside the mortar induced by higher luffa fiber content or longer lengths. As a result, the flow of aggregates will not only hindered but also cement slurry will be absorbed, thereby reducing the flowability of mortar.

### 3.2 Analysis of compressive strength

**Fig 4:**
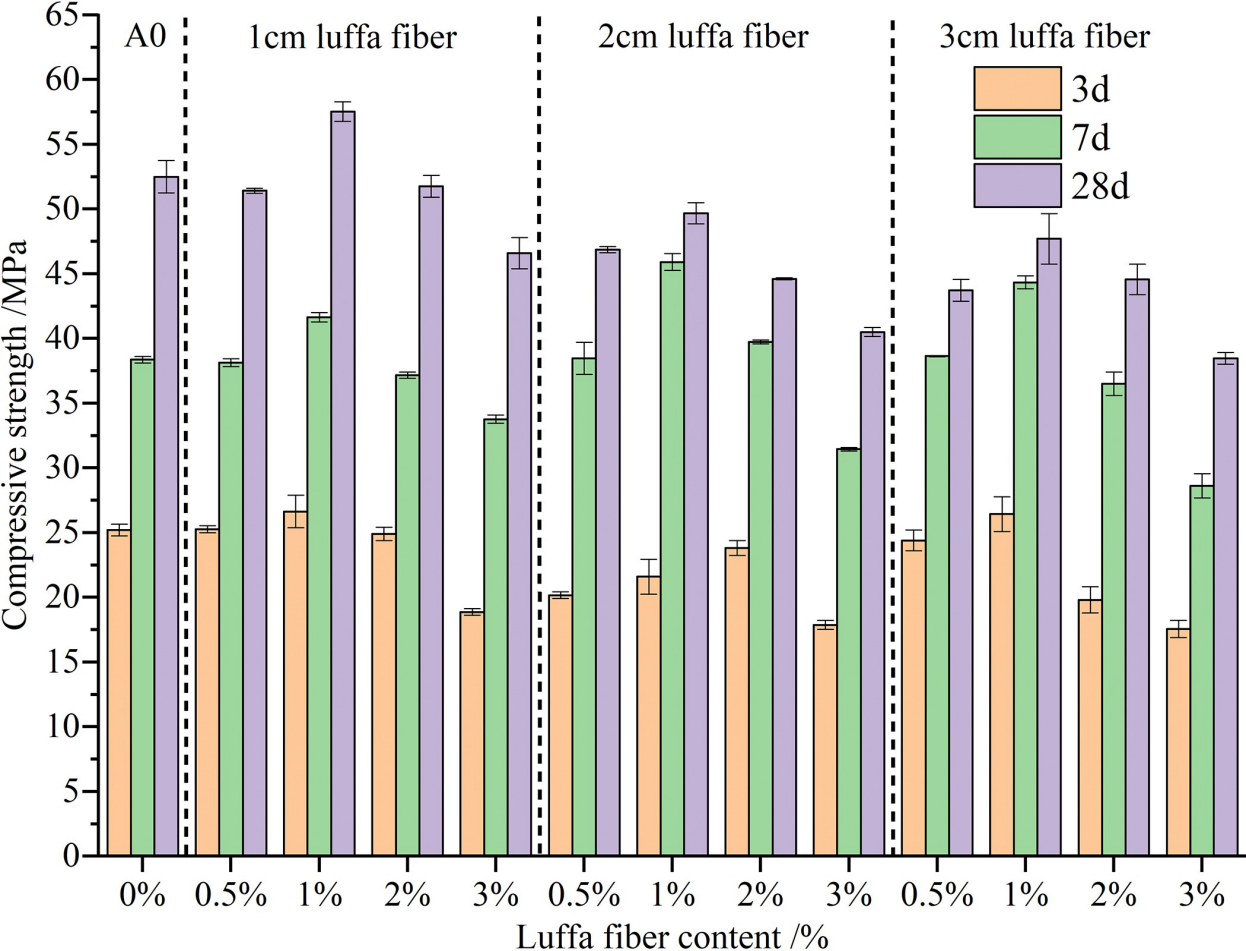
Compressive strength of luffa fiber reinforced cement mortar at different ages.

Fig 4 shows the impact of different content and length of luffa fibers on the compressive strength of cement mortar at different ages. The experimental results showed that when the content of luffa fiber was 0.5% or 1%, the compressive strength of cement mortar exhibited an increasing trend. On the contrary, when the content of luffa fiber exceeded 1%, the compressive strength showed a decreasing trend. This pattern is similar to Anandaraj’s research results, where a combination of 1% luffa fiber and 25% rice husk ash (RHA) can provide the best compressive, cracking, and bending strength [19]. When the content of luffa fiber was 1% and the length was 1 cm, the compressive strength of cement mortar at each age was the highest, reaching 57.63 MPa at 28 days, an increase of 10.81% compared to ordinary cement mortar. When the length of luffa fiber increased to 2 cm and 3 cm, the compressive strength at 28 days decreased by 4.87%% and 14.81% compared to ordinary cement mortar, respectively. Increasing the fiber content of luffa fiber to 3%, with luffa fiber lengths of 1 cm, 2 cm, and 3 cm respectively, resulted in a decrease in 28 days compressive strength of ordinary cement mortar by 26.2%, 38.5%, and 41.95%. This result can be explained by considering that the existence of more fibers per unit volume yields a better crack-limiting enhancement effect of the fibers. However, when the luffa fiber content was too high, the water absorption of the luffa fiber reduced the binding water in the matrix. At the same time, the resistance between fibers increased, leading to a decrease in the slump of the mortar and a deterioration in workability, ultimately resulting in decreased strength [39]. Moreover, an excess of fibers leads to the generation of more pores, reducing the density of the mortar and consequently decreasing its compressive strength. As can be seen, with a certain amount of luffa fiber content, the compressive strength of cement mortar was higher when the size of the luffa fiber was 1 cm. As the length of the luffa fiber increased to 2–3 cm, the compressive strength of the mortar gradually decreased. This is because as the fiber length increases, the possibility of "agglomeration" in the cement mortar matrix greatly increases. Many agglomerated fibers form fiber balls, making the specimen porous. As a result, both the density and strength are significantly reduced.

### 3.3 Analysis of flexural strength

**Fig 5:**
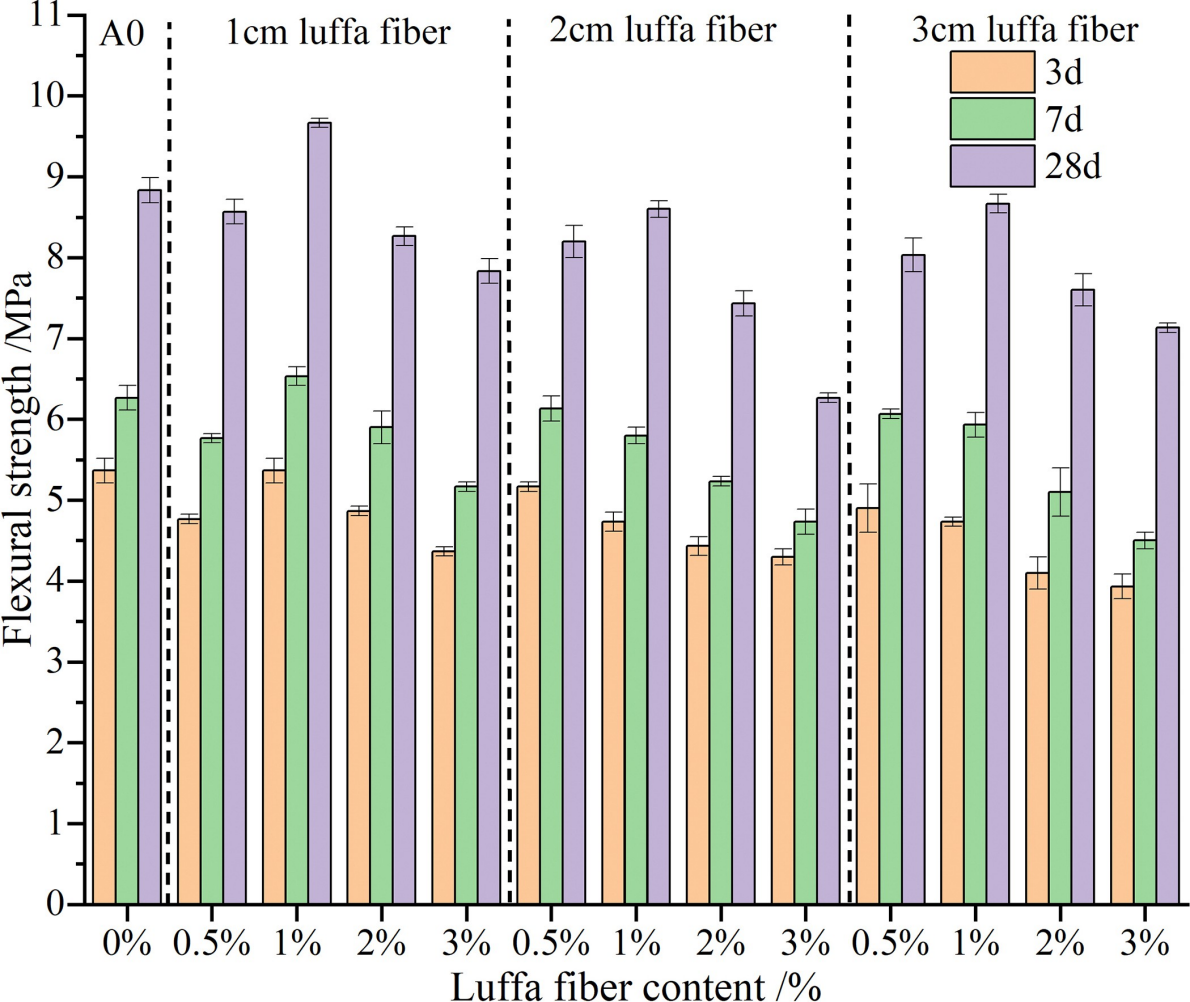
Flexural strength of luffa fiber reinforced cement mortar at different ages.

Fig 5 shows the impact of different content and length of luffa fibers on the flexural strength of cement mortar at different ages. The experimental results showed that the flexural strength of cement mortar increased with the addition of luffa fiber. Nonetheless, the magnitude of the increase varies with the amount and length of fibers added. When the content of luffa fiber was 1% and the length was 1 cm, the maximum flexural strength at 28 days can reach 9.68 MPa, which is 9.47% higher than ordinary cement mortar. This is because the appropriate amount and length of luffa fiber play a "bridging" role in the mortar. When the mortar was loaded and micro cracks occurred inside, the matrix transferred stress to the fibers. The fibers were pulled out or broken from the matrix by debonding with the matrix, consuming a large amount of energy, and thereby improving the flexural strength of the cement mortar. When the content of luffa fiber was 0.5%, 2%, and 3% respectively, it had a negative impact on the flexural strength of the cement mortar. Especially when the content of luffa fiber reached 3% and the length of luffa fiber was 3 cm, the flexural strength at 28 days decreased by 1.8 MPa compared to ordinary cement mortar. The length of luffa fibers has a similar impact on the flexural and compressive strength of cement mortar. The flexural strength of the cement mortar decreased with the increase in the fiber length. When the length of luffa fibers was 1 cm, the flexural strength of cement mortar was better. This is because the existence of a longer luffa fiber facilitates the mortar to tangle and form clumps during mixing, resulting in uneven dispersion and adverse effects on the flexural strength of the cement mortar.

### 3.4 Shrinkage performance analysis

**Fig 6:**
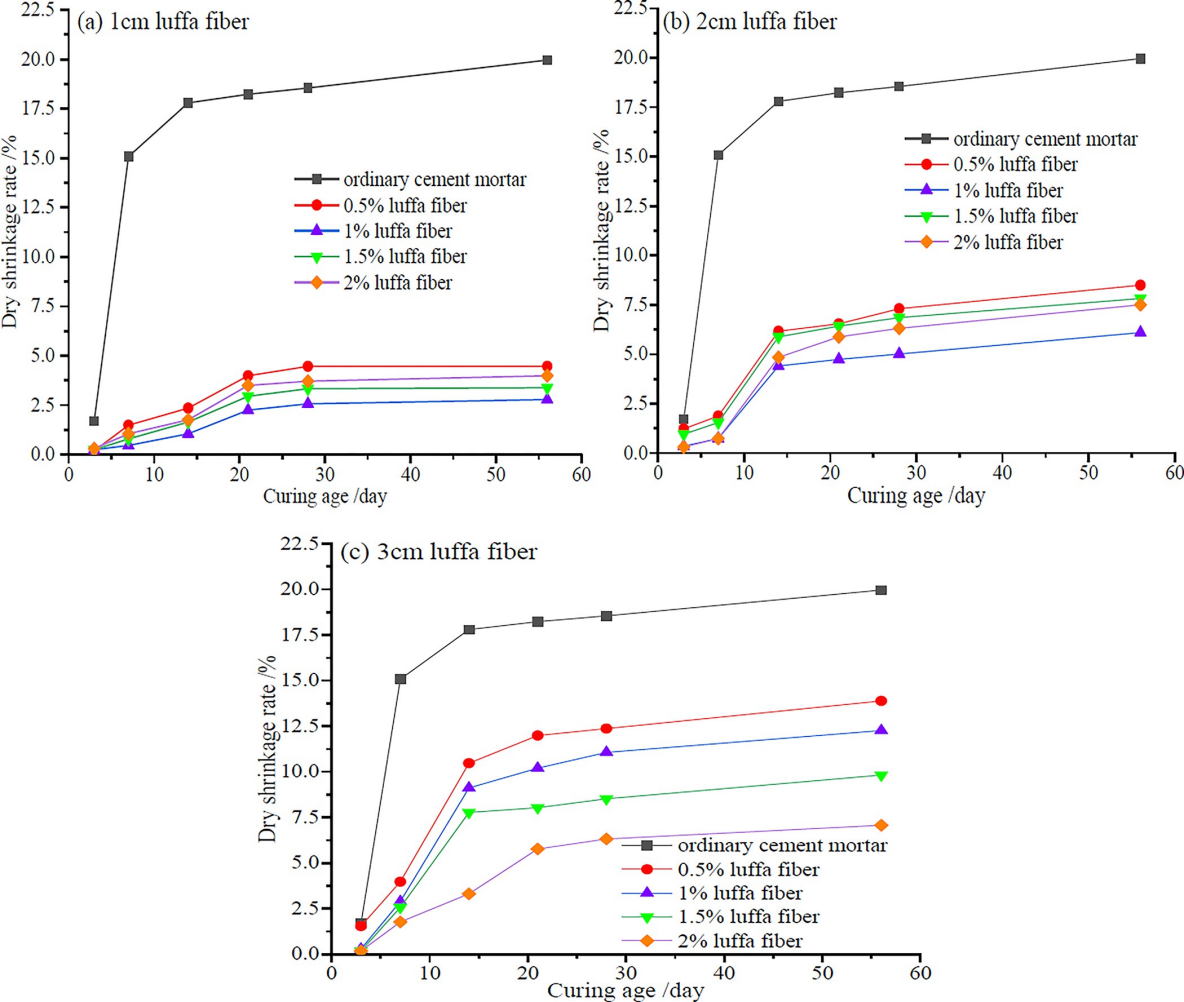
Dry shrinkage rate of luffa fiber reinforced cement mortar at different ages.

Based on JC/T 603–2004 "Standard Test Method for Drying Shinkage of Mortar", the drying shrinkage temperature is 20°C± 1°C and the relative humidity is 50% ± 4%. The length of luffa fiber reinforced cement mortar specimens at different ages was tested, and the corresponding dry shrinkage rate was calculated [37]. The (a)-(c) in Fig 6 represent the trend of drying shrinkage of mortar with different luffa fiber contents of 1, 2, and 3 cm, respectively, as a function of age. As can be seen, whether it is ordinary cement or luffa fiber reinforced cement mortar, the dry shrinkage rate increased with age in the early stage and gradually flattened in the later stage. This is mainly due to insufficient hydration of early cement, resulting in a higher water content in the capillary pores inside the matrix, leading to faster water evaporation. As the age increases, the external environment invades the water to reach equilibrium, and the drying shrinkage of the mortar becomes slow [35].

As can be ascertained from Fig 6, due to the ability of luffa fiber to store free water, more free water participates in cement hydration inside the luffa fiber cement mortar, resulting in a higher ability to resist dehydration shrinkage. Therefore, the addition of different lengths of luffa fiber can significantly improve the drying shrinkage performance of the mortar. However, the influence of different lengths on the drying shrinkage rate of the mortar is also different. The best effect on improving the dry shrinkage of cement mortar was recorded when the length of the luffa fiber was 1 cm. In striking contrast, the effect on improving the dry shrinkage of cement mortar was poor when the length of the luffa fiber was 3 cm. Additionally, the fiber content has a significant impact on the dry shrinkage rate of mortar at different lengths of luffa fibers. When the length of luffa fibers was 1 cm and the content was 1%, the dry shrinkage rate of mortar at different ages significantly decreased, with 56 days dry shrinkage rate of 2.78%, which was 7.17 times lower than that of ordinary cement mortar. From Fig 6(B), it can be seen that when the length of the luffa fiber was 2cm and the content was 1%, the dry shrinkage rate of the mortar significantly decreased, with a shrinkage rate of 6.09% at 56 days, which was 3.27 times lower than that of ordinary cement mortar. Fig 6(C) indicates that when the length of the luffa fiber was 3 cm, the dry shrinkage rate continuously decreased with the increase of the fiber content. When the luffa fiber content was 2%, the dry shrinkage rate was 7.07%, which was 2.81 times lower than that of ordinary cement mortar. This is because when the fibers of the luffa are longer, the bonding area between the fibers and the matrix is larger. Consequently, the range of stress transmission is wider. However, if the fibers are too long, they are prone to entanglement and clumping, making it difficult to achieve uniform dispersion in the mortar matrix. In addition, when the fiber content is small, it can have a good crack limiting effect. When the content is large, long fibers are prone to entanglement and clumping. When the induced negative effect is greater than the positive effect, the dry shrinkage rate of cement mortar will be increased instead of decreased. Therefore, when the fiber content of luffa fiber is 1% and the length is 1–2 cm, it can effectively reduce the drying shrinkage of cement mortar.

### 3.5 Load-deflection analysis

**Fig 7:**
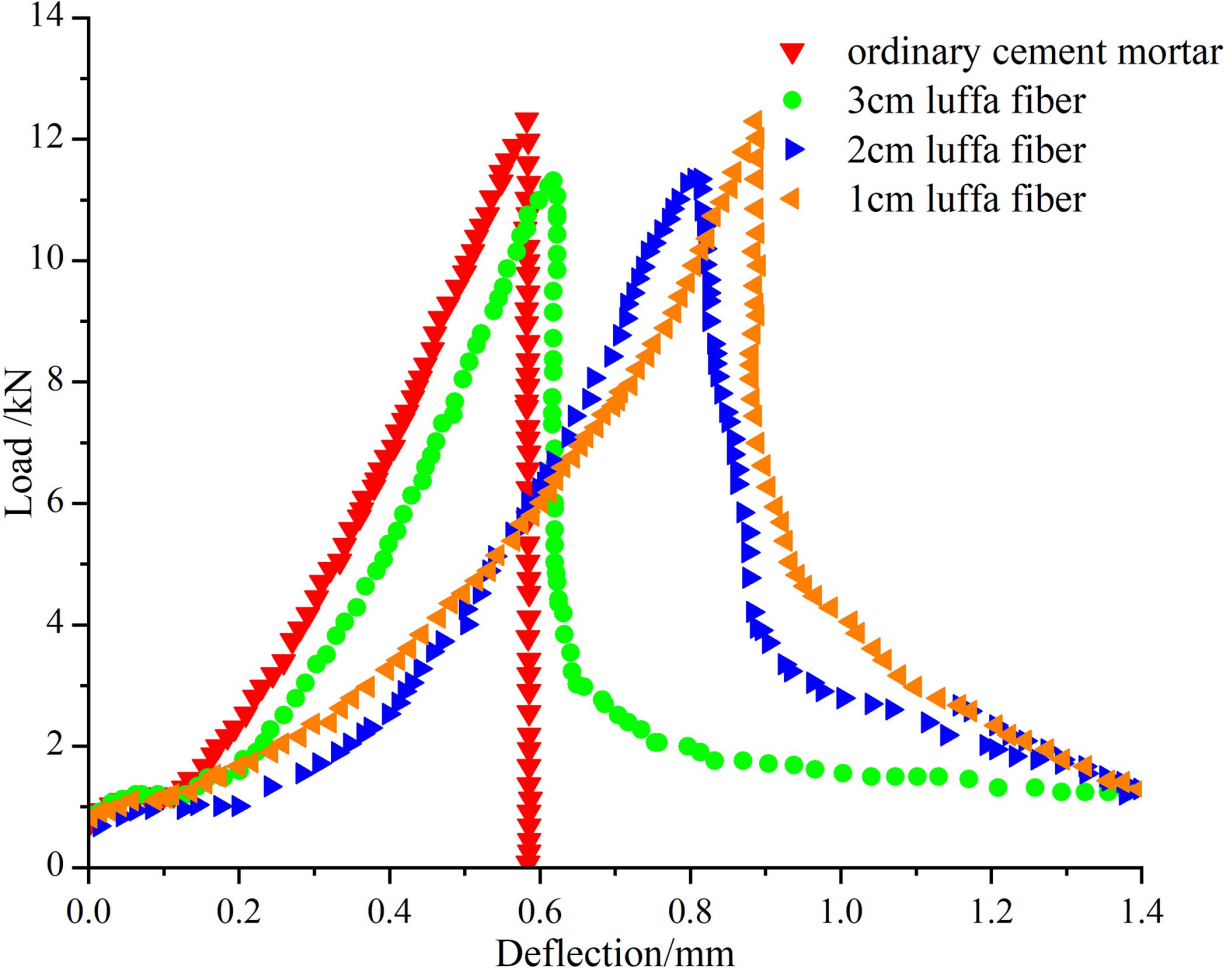
Load-deflection curves of ordinary cement mortar and luffa fiber cement mortars.

The experiment showed that when the content of luffa fiber was 1%, the flexural and compressive strength of cement mortar was higher. Therefore, referring to the requirements of ASTM C1018 in the United States [38], the four point loading method was used to conduct bending load-deflection tests on cement mortar with a 1% content of luffa fiber and lengths of 1 cm, 2 cm, and 3 cm, respectively. The results were compared with ordinary cement mortar. As can be observed in Fig 7, it was found that ordinary cement mortar rapidly failed when it reached its ultimate bearing capacity. The failure process of luffa fiber reinforced mortar showed a parabolic trend, with the initial and later deflections exhibiting an increasing and decreasing trend with the increase of the load, respectively. When the content of luffa fibers was 1%, the different lengths of luffa fibers can improve the bearing capacity of cement mortar. As a result, the deformation of cement mortar under peak load increases, reflecting that luffa fibers can enhance the bending performance of cement mortar. When the fiber length of the luffa was 1 cm, the cement mortar specimen exhibited a significantly higher deflection than other groups, and at this time, the toughness of the cement mortar was the strongest, with a significant increase in peak load. When the length of luffa fibers in cement mortar was 2 cm, the load-deflection curve of the mortar was greater than that of ordinary cement mortar without fibers added. In the test group with a length of 3 cm of luffa fibers in mortar, the peak load was much lower than that of other groups. When the specimen was subjected to the peak load, the failure rate of the specimen was fast, and the curve showed a rapid downward trend. From a macro perspective, the expansion of cracks in ordinary cement mortar during bending loading takes place in an unstable state. When the expansion tension generated during the crack extension process is greater than the resistance or closing force of the crack tip material, the failure of the mortar specimen will turn into an unstable failure. The incorporation of 1–3 cm of luffa fiber can play a bridging role in cement mortar, slow down the expansion trend of cracks, increase the peak deflection of the mortar, and absorb more energy during the expansion process. Thereby, the toughness of cement mortar can be significantly improved.

### 3.6 SEM-EDS analysis

**Fig 8:**
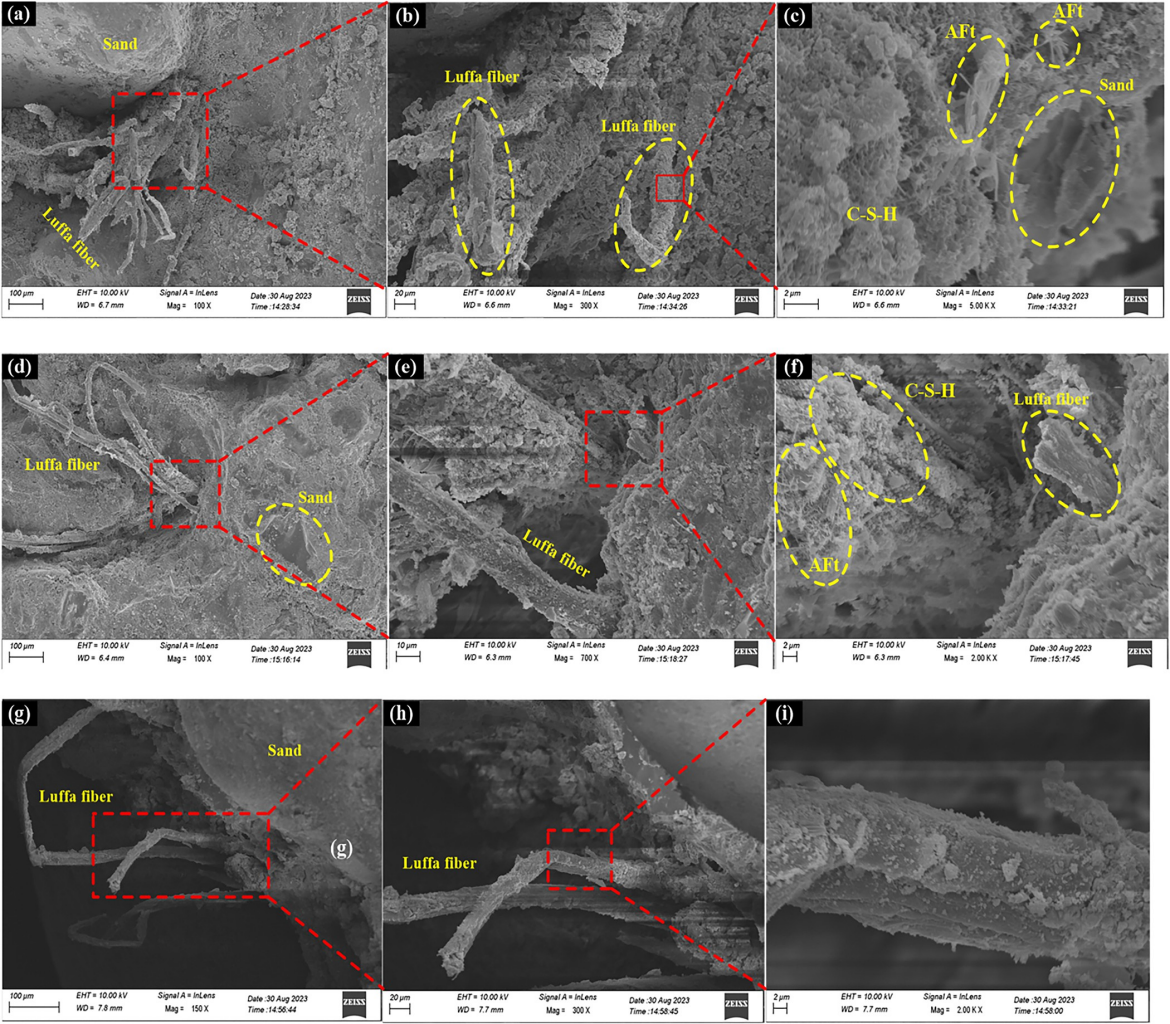
SEM images of cement mortar with different lengths of luffa fibers.

Fig 8 shows the SEM images of cement mortar with a 1% content of luffa fibers and lengths of 1 cm, 2 cm, and 3 cm, respectively. As can be seen from Fig 8(A)–8(C), when the length of the luffa fiber was 1 cm, the luffa fiber connected with the cement slurry and sand and formed a dense network structure. When the luffa fiber reinforced cement mortar in Fig 8(A) was magnified to 20 um, it was found that the surface of the luffa fiber was covered with a large number of irregular pommel particles (Fig 8(B)). Further magnification of the irregular pommel particles on the surface of the luffa fiber is shown in Fig 8(C). It can be seen that the irregular pommel particles on the surface of the luffa fiber are mainly tinfoil-shaped C-S-H (hydrated calcium silicate), needle-rod shaped AFt (ettringite) and sand. The above result indicates that when the length of the luffa fiber is 1 cm, there is a large amount of C-S-H (hydrated calcium silicate) and AFt (ettringite) on the surface of the luffa fiber. The latter can be better fused with cement mortar to form a dense system and induces the development of a certain mechanical biting force, playing a reinforcing role. Thus, the bending, compressive strength, and toughness of cement mortar were improved. From Fig 8(D)–8(F), it can be seen that when the length of the luffa fiber was 2 cm, there was a small number of pores between the luffa fiber and the cement slurry. When the mortar around the luffa fiber was further enlarged to 2 um, it was found that there was still a small number of tinfoil-like C-S-H (hydrated calcium silicate) and needle-rod like AFt (ettringite) around the luffa fiber. As can be ascertained from Fig 8(G)–8(I), when the length of the luffa fiber was 3 cm, there is a phenomenon of separation between the luffa fiber and the cement slurry. Further magnification of the surface of the luffa fiber is shown in Fig 8(H)–8(I), where it can be seen that the surface of the luffa fiber is almost anhydrous. This is due to the long length of the luffa fiber, which causes bending and winding effects, reducing the uniform dispersion in its cement mortar matrix, and even hindering the generation of hydration products in the mortar. At the same time, stress concentration can be also caused, leading to a decrease in the mechanical properties of cement mortar.

**Fig 9:**
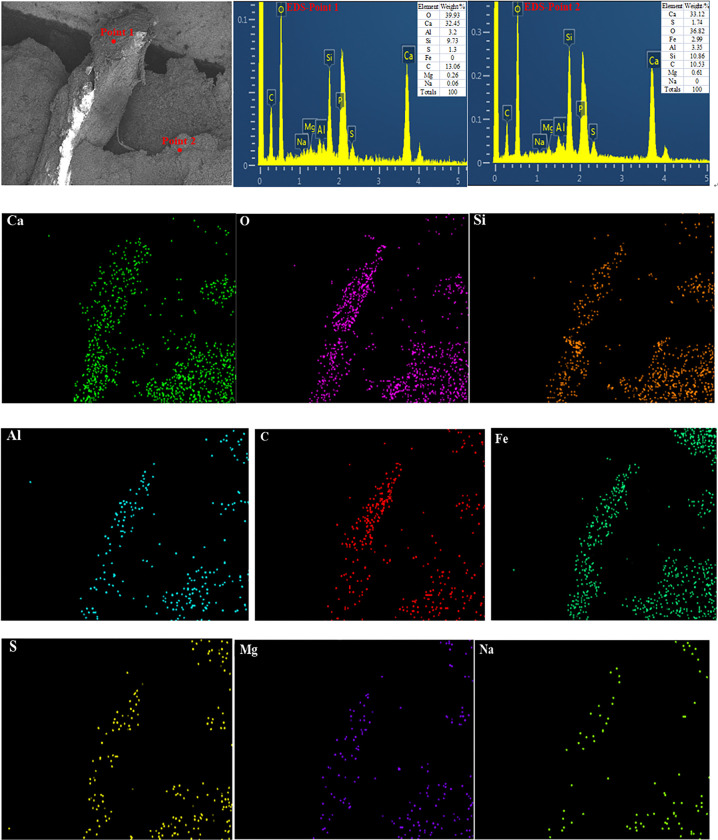
EDS and Mapping diagram of cement mortar with different lengths of luffa fiber.

Fig 9 shows the energy spectrum analysis EDS and Mapping of luffa fiber reinforced cement mortar. As can be seen, the main elements on and around the surface of luffa fiber are O, Ca, Al, Si, S, Fe, and C, especially O, Ca, Al, and Si, which account for a high proportion. Hence, it can be argued that luffa fiber is well bonded to the surface of cement mortar, and there may be C-S-H (hydrated calcium silicate), C-A-H (calcium aluminate hydrate) gel on the surface of luffa fiber. It can also be seen from the mapping image that the elements O, Ca, Al and Si are closely distributed and cross each other, which indicates that the combination between the surface of luffa fiber and cement mortar is good. In addition, gel is generated, which can improve the mechanical properties of cement mortar.

### 3.7 CT tomography analysis

**Fig 10:**
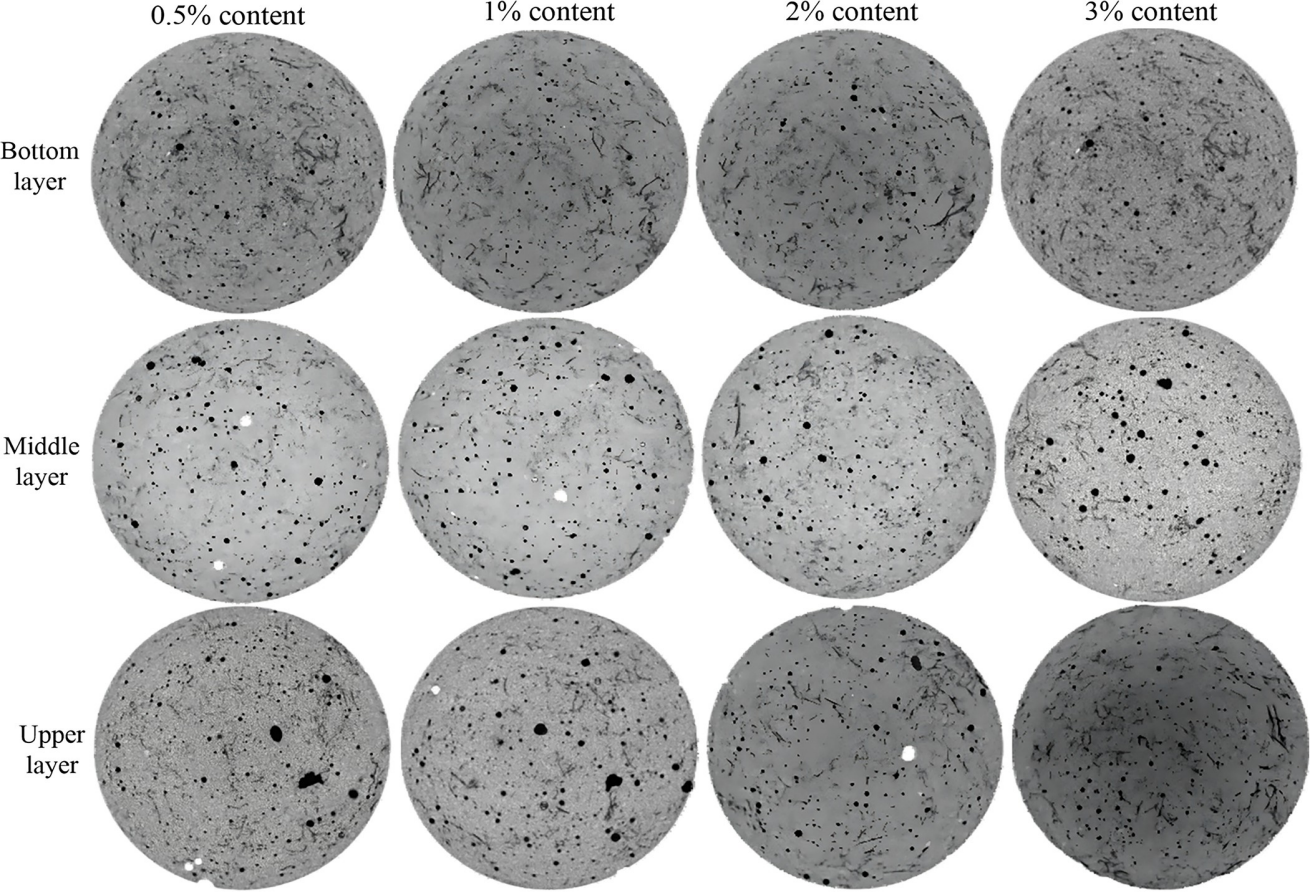
CT scanning of cement mortar with different content of luffa fiber.

Fig 10 shows the CT scan images of cylindrical cement mortar with a length of 1 cm and a dosage of 0.5%, 1%, 2%, and 3%, respectively. As can be seen, a large number of luffa fibers gather at the top and bottom of the specimen, with a smaller distribution in the middle. This is because. during the vibration process, smaller fibers will float up with the cement mortar and gather at the top of the specimen. At the same time, some luffa fibers will be also accumulated at the bottom of the specimen as the coarser mortar sinks. It can also be observed that with the increase of the content of luffa fibers, the corresponding pores also increase and an agglomeration phenomenon occurs. This is because the increase in the fiber content requires more fibers wrapped in cementitious materials. The incomplete wrapping of luffa fibers deteriorates their internal compactness, and agglomeration and pore phenomenon occur.

**Fig 11:**
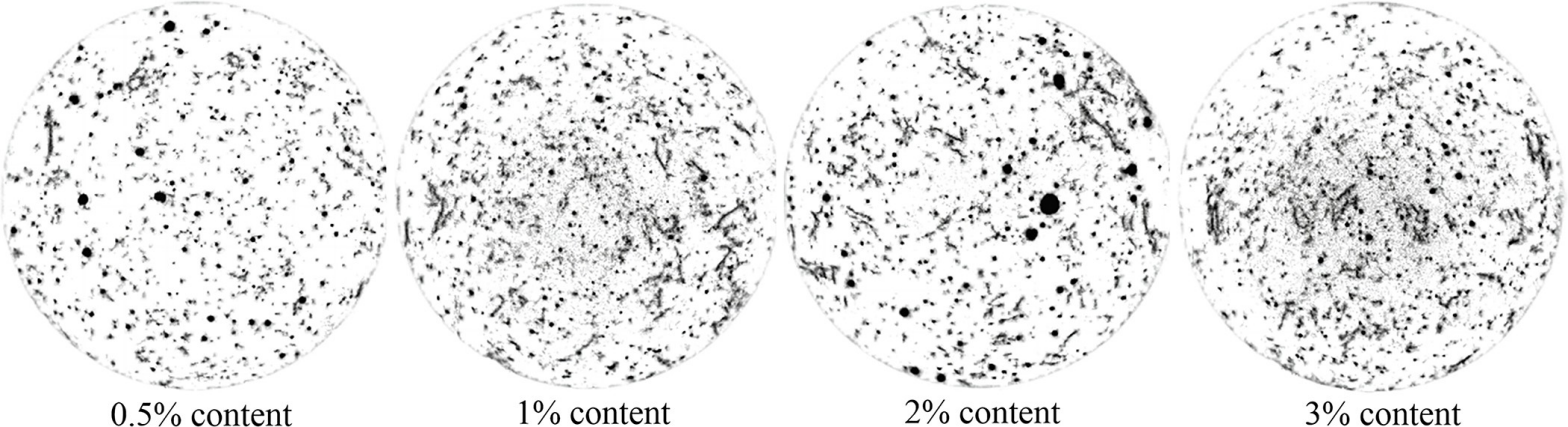
2D segmentation of pores and fibers in cement mortar with different content of luffa fibers.

**Fig 12:**
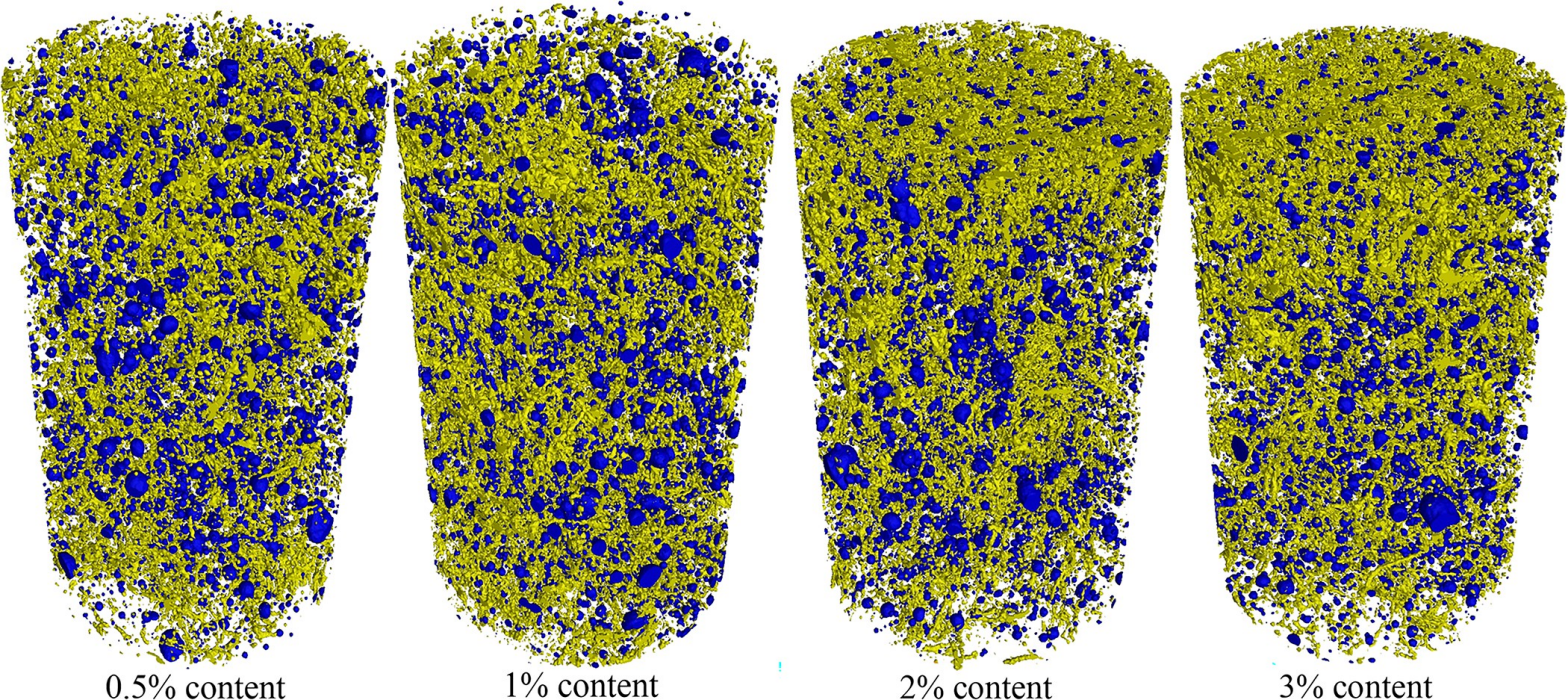
3D segmentation of pores and fibers in cement mortar with different content of luffa fibers.

To further observe the state of luffa fibers inside cement mortar, Dragonfly software was used to segment and visualize CT images in 3D, as shown in Figs 11 and 12. As can be seen, when the content of luffa fibers was 0.5%, the overall number of luffa fibers in the specimen was not large. In addition, luffa fibers were gathered at the top and bottom of the specimen. Thus, only a small number of fibers can participate in the stress in the middle of the specimen, making the bridging effect of the fibers unable to be fully exerted. When the content of luffa fiber was 1%, although a phenomenon of fiber aggregation at the top and bottom of the specimen took place, the overall distribution of fibers was relatively uniform and there were fewer pores. The weak layer in the middle of the specimen has been improved compared to 0.5% content. When the content of luffa fiber was 2% and 3%, although there was a large amount of fiber distributed in the middle of the specimen, a thick fiber aggregation layer appeared at the top and bottom of the specimen. This aggregation layer seriously hinders the bonding effect of the cementitious material between the sand, and at the same time, the number of pores also increases, which affects the mechanical properties of the cement mortar. This effect could explain why the continuous reduction in the mechanical strength of the cement mortar when the content of luffa fiber exceeded 1%.

## 4. Conclusions

A 2% NaOH pre-treatment of luffa fiber was introduced into cement mortar to investigate the influence of luffa fiber length and content on both the mechanical and microscopic properties. The study yielded the following findings:

1. When the content of luffa fiber was 1% and the length was 1 cm, the 28-day compressive strength and flexural strength of the cement mortar were 57.63 MPa and 9.68 MPa, respectively, which represent an increase of 10.81% and 9.47% compared to the 28-day compressive strength (52.5 MPa) and flexural strength (8.83 MPa) of the standard cement mortar. When the content of luffa fiber was 1% and the lengths were 1 cm and 2 cm, the 56-day drying shrinkage rates of the cement mortar were 2.78% and 6.09%, respectively, corresponding to a reduction of 7.17-fold and 3.27-fold compared to those of the standard cement mortar. Additionally, when the fiber content was 1% and the length was 1–3 cm, the load-deflection behaviour of luffa fiber cement mortar was notably superior to that of standard cement mortar.
2. Through SEM-EDS and mapping analysis, it was observed that when the length of the luffa fiber was 1 cm, a significant presence of C-S-H and AFt was detected on the fiber’s surface. This effect facilitated the integration with the cement mortar, forming a dense system and providing secondary reinforcement, consequently improving the compressive, flexural strength, and toughness of the cement mortar. However, with an increase in luffa fiber length to 2–3 cm, the possibility of "agglomeration" within the cement mortar matrix raised, and the gel formed on the luffa fiber’s surface diminished.
3. CT scanning revealed that a substantial concentration of luffa fibers was clustered at the top and bottom of the specimen, with less even distribution in the middle. As the luffa fiber content increased, the voids and fiber aggregation correspondingly increased. At a 1% luffa fiber content, although there was some fiber aggregation at the specimen’s top and bottom, the overall fiber distribution remained relatively uniform. This resulted in an improved weak layer in the middle of the specimen compared to a 0.5% content, allowing for the full utilization of the bridging effect of the fibers.

In conclusion, at a loofah fiber content of 1% and a length of 1 cm, these fibers can act as a "bridge" and reinforcement within cement mortar. This significantly improves the toughness, flexural strength, shrinkage resistance, and agglomeration ability of the cement mortar. However, there are also limitations considering that the impact of freeze-thaw resistance, alkaline corrosion resistance, sulfate resistance, energy absorption under load, fiber orientation angle, and critical fiber volume fraction of luffa fiber cement mortar was not taken into account. Addressing these issues will form the direction of future research, with the aim of applying luffa fiber-cement mortar in building construction and road construction.

## Supporting information

S1 File The raw data of study. (DOCX)
