# Supplementary material for: Mechanical properties and microstructure of pre-treated luffa fiber reinforced cement mortar
Source: PLoS One. 2025 Feb 7;20(2):e0314213. doi: 10.1371/journal.pone.0314213 (PMC11805437; doi:10.1371/journal.pone.0314213)
Supplement: S1 File — (DOCX) [file pone.0314213.s001.docx]

The Raw Data of Study

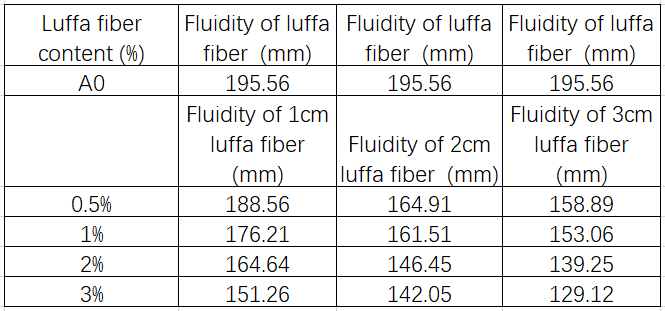


Figure 3a Luffa fiber mortar flowability

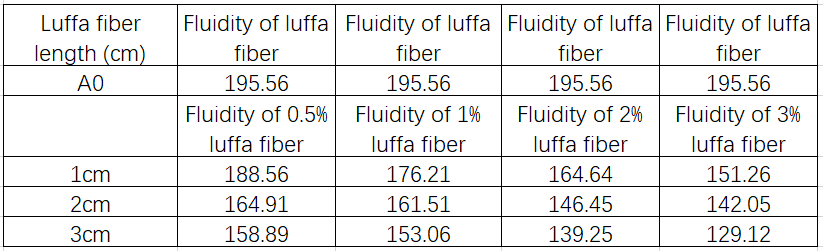


Figure 3b Luffa fiber mortar flowability

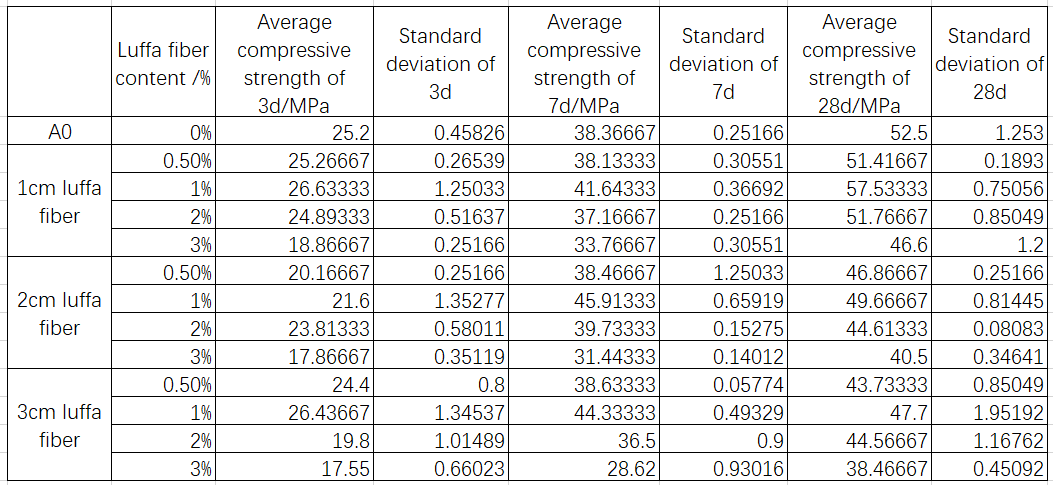


Figure 4 Compressive strength of luffa fiber reinforced cement mortar at different ages

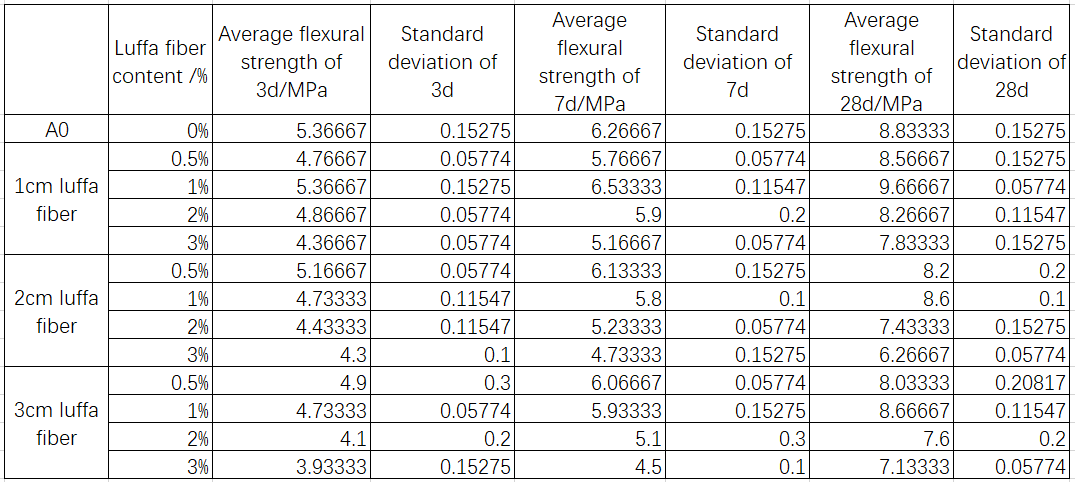


Figure 5 Flexural strength of luffa fiber reinforced cement mortar at different ages


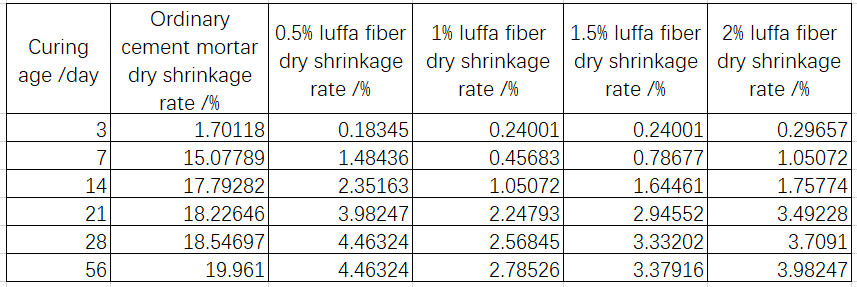


Figure 6a Dry shrinkage rate of luffa fiber reinforced cement mortar at different ages

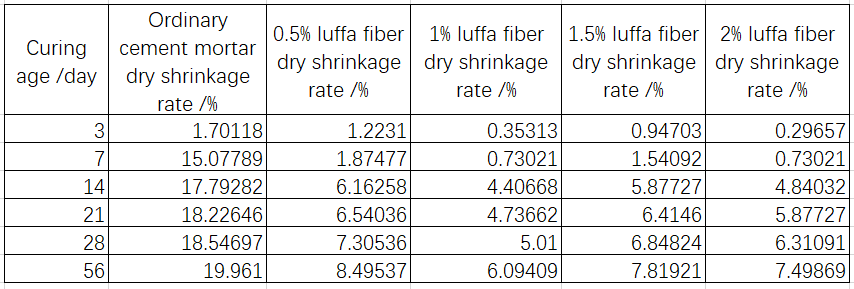


Figure 6b Dry shrinkage rate of luffa fiber reinforced cement mortar at different ages

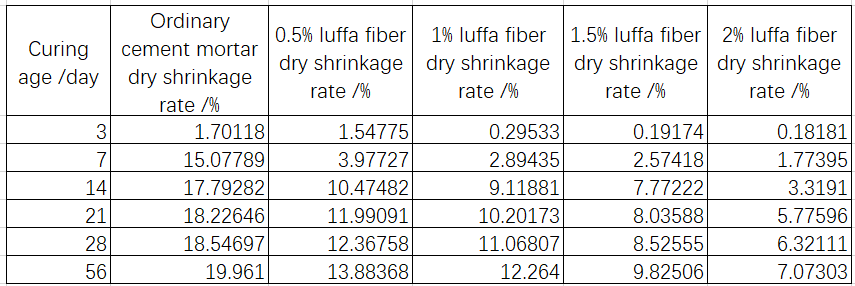


Figure 6c Dry shrinkage rate of luffa fiber reinforced cement mortar at different ages
